# From lockdown to recovery: changing patterns of viral infection severity in a pediatric cohort with asthma

**DOI:** 10.3389/falgy.2025.1645968

**Published:** 2025-09-24

**Authors:** Cassidy Jones, Matthew Laws, Shahwar Yousuf, Andrew Delo, Susanna Hartzell, Emma Kinder, Ashton Ingold, Bobby L. Boyanton, Dana Frederick, Rachel A. Frenner, Erin Hathorn, Peter M. Mourani, Joshua L. Kennedy

**Affiliations:** 1 Arkansas Children’s Research Institute, Little Rock, AR, United States; 2 Department of Internal Medicine, College of Medicine, University of Arkansas for Medical Sciences, Little Rock, AR, United States; 3 Department of Pediatrics, University of Arkansas for Medical Sciences, Little Rock, AR, United States; 4 Department of Pathology, University of Arkansas for Medical Sciences, Little Rock, AR, United States; 5 Department of Pathology, Arkansas Children’s Hospital, Little Rock, AR, United States; 6 Department of Bioinformatics, Arkansas Children’s Hospital, Little Rock, AR, United States; 7 Department of Internal Medicine, University of Arkansas for Medical Sciences, Little Rock, AR, United States

**Keywords:** asthma, viral infections, disease severity, pediatric asthma, children

## Abstract

**Background:**

Respiratory viruses such as rhinovirus and respiratory syncytial virus (RSV) are common triggers of asthma exacerbations in children. The COVID-19 pandemic introduced non-pharmaceutical interventions (NPIs) that altered viral circulation; however, their long-term effects on pediatric asthma outcomes remain unclear.

**Objective:**

To evaluate how the epidemiology and severity of respiratory viral infections in children with asthma changed before, during, and after COVID-19-related NPIs.

**Methods:**

We conducted a cross-sectional analysis of pediatric asthma patients (ages 4–18) with laboratory-confirmed respiratory viral infections from 2018 to 2024 at Arkansas Children's (AC) and AC Northwest (ACNW). Viral detection was performed using the BioFire® Respiratory Panel. Clinical severity was evaluated using a modified World Health Organization Ordinal Scale for Clinical Improvement (mWHO OSI). Patients were categorized by period (pre-NPI, NPI, post-NPI), viral type, rurality, and Childhood Opportunity Index (COI).

**Results:**

This study included 9,391 pediatric asthma patients with laboratory-confirmed viral infections. RV/EV was the most common virus during all periods. Viral incidence decreased during NPIs but rebounded post-NPI with unusual seasonality. mWHO OSI scores declined over time (pre-NPI: 2.98; NPI: 2.49; post-NPI: 2.28), with significant reductions in hospitalizations, PICU admissions, and oxygen use (*p* < 0.0001). Severe disease (mWHO OSI 6–8) was infrequent. Rural and low-COI patients exhibited higher severity, although disparities narrowed post-NPI.

**Conclusions:**

NPIs were associated with sustained reductions in asthma-related illness severity, even with increased viral detection post-pandemic. These findings highlight the long-term impact of public health measures on pediatric asthma outcomes and emphasize the need for ongoing surveillance of respiratory viruses and health disparities.

## Introduction

Asthma is one of the most common chronic diseases, affecting approximately 334 million people worldwide (1). This serious respiratory condition imposes a significant burden on individuals and healthcare systems, leading to a decreased quality of life and, in some cases, premature death. Although asthma affects individuals across all age groups, adolescents experience the highest prevalence and severity of the disease (2).

According to the Centers for Disease Control and Prevention (CDC), the national prevalence of asthma attacks among children (aged 18 years and under) with current asthma is 1,811,063 (38.7%) (3). Viral infections are known triggers for asthma exacerbations, with rhinovirus and RSV being the two most common contributors (4–11). Other viral pathogens may also play a significant role in asthma exacerbations (12), although their specific mechanisms and contributions remain less well understood.

The COVID-19 pandemic dramatically transformed the landscape of respiratory viral infections and asthma exacerbations in the pediatric population (13, 14). NPIs implemented to curb the spread of SARS-CoV-2 also resulted in a decrease in the number of acutely ill children presenting to emergency departments (15). Early in the pandemic, the frequency of weekly asthma exacerbations declined by 50%, coinciding with the widespread implementation of NPIs (14). Reports indicate an absence of most respiratory viruses during their peak seasons, except for adenovirus (ADV) and respiratory syncytial virus (RSV) (16). These observations raise important questions about the relationship between viral circulation, NPIs, and asthma outcomes. While NPIs reduced the transmission of SARS-CoV-2, they also disrupted the seasonal patterns of respiratory viruses and bacterial infections. Understanding how these interventions influenced viral epidemiology and asthma severity is vital for future public health strategies.

Given the established link between respiratory infections and asthma exacerbations, it is essential to investigate the impact that NPIs have had on the epidemiology of specific viral infections and their contribution to asthma exacerbations. We aim to provide valuable insights into the interplay between viral infections, asthma severity, and the broader impact of NPIs on respiratory health. The objective of this study is to determine whether the implementation of NPIs during the COVID-19 pandemic changed the epidemiology of respiratory viruses in children with asthma and whether these changes affected clinical severity over time.

## Methods

### Study design and population

This cross-sectional study was approved by the Institutional Review Board (IRB) of the University of Arkansas for Medical Sciences, with a waiver for consent and HIPAA authorization (IRB 262416 and 274080). The study population included patients aged 4- to 18-years who presented with a positive viral test and a diagnosis of asthma at Arkansas Children's (AC) (Little Rock, AR) or Arkansas Children's Northwest (ACNW) (Springdale, AR). The included children also had to reside in Arkansas. The Arkansas Children's Health System is the only pediatric health system in the state and serves almost exclusively as the pediatric referral center for the State of Arkansas and parts of surrounding states. We selected the 4- to 18-year age range to ensure greater diagnostic certainty, as children under 4 years are less likely to have a confirmed asthma diagnosis due to overlapping clinical features with other respiratory illnesses. These patients were evaluated in outpatient clinics, emergency departments (ED), inpatient wards, or the Pediatric Intensive Care Unit (PICU). Patients were also screened for viral infections during pre-operative assessments.

Asthma diagnoses were identified using International Classification of Diseases, 10th Revision (ICD-10) codes (J45*). The AC clinical pathology laboratory identified samples from all pediatric patients who tested positive for a respiratory pathogen between January 2018 and December 2024. Viral testing was performed for clinical purposes using the BioFire® FilmArray® Respiratory Pathogen Panel (RPP; bioMerieux, Durham, NC, USA) from January 1, 2018, to December 31, 2024, at AC or ACNW. Although data were available from November 2017 through January 2025, we limited the analytic window to full calendar years (2018–2024) to ensure consistent seasonal comparisons. The RPP detected 21 respiratory pathogens, including ADV, coronaviruses (COV), human metapneumovirus (HMPV), rhinovirus/enterovirus (RV/EV), influenza A (INF A), influenza B (INF B), parainfluenza viruses (PIV), RSV, *Bordetella pertussis*, *Bordetella parapertussis*, *Chlamydia pneumoniae*, and *Mycoplasma pneumoniae*. In June 2020, the U.S. Food and Drug Administration approved a newer version of the RPP for clinical use, which included SARS-CoV-2, bringing the total tested by the RPP to 22. For this study, children with positive tests for *Bordetella pertussis*, *Bordetella parapertussis*, *Chlamydia pneumoniae*, and *Mycoplasma pneumoniae* were excluded.

We included one other viral testing platform. The Xpert® Xpress Cov-2/Flu/RSV (4-plex) (Cepheid, Sunnyvale, CA, USA) was introduced in December of 2021 and included testing for INF A, INF B, RSV, and SARS-CoV-2. We excluded patients who were tested only for SARS-CoV-2. A yearly breakdown of testing platforms used at our facilities revealed that the RPP constituted 100% of testing prior to 2020 and has offered comprehensive detection of respiratory viruses across all periods, with usage between 34% and 100% at our facilities from 2017 to 2024. After the introduction of the 4-plex, this test was used between 25% and 32% in our facilities.

Individuals who tested positive for the same viral infection within 30 days were excluded. However, if another virus was identified, either as a coinfection or as a new infection, then they were not excluded, even if this virus was detected within 30 days of the original positive test.

### Data collection

Data were extracted from the electronic medical record (EPIC, Verona, WI, USA) using Structured Query Language (SQL). The extracted data included demographics (age, sex, race, and ethnicity), RPP results, zip codes, collection date, and comorbidities (e.g., cancer, sickle cell disorders, gastrostomy status, corrected congenital heart malformations, asthma, seizure disorders, and obesity). Hospitalization status was categorized by care setting (e.g., outpatient clinic, ED, inpatient ward, PICU). Severity scale indicators for the modifed World Health Organization Ordinal Scale for Improvement (mWHO OSI) were recorded (e.g., oxygen flow status [high ≥ 6l or low < 6l], non-invasive ventilation, mechanical ventilation, pressor support, Extracorporeal Membrane Oxygenation [ECMO], Renal Replacement Therapy [RRT], and mortality).

The data for the Rural-Urban Commuting Area (RUCA) classification (U.S. Department of Agriculture, Economic Research Service [USDA, ERS], 2010) and the Child Opportunity Index (COI) (diversitydatakids.org, 2023, “Child Opportunity Index 2.0 Zip Code data”) were included for each participant. The RUCA classification categorized subjects into RUCA 1–4 (more urban) and 5–10 (more rural). The COI data divided subjects into three groups: very low/low, moderate, and high/very high opportunity.

### Symptoms severity scoring

Based on the chart review, a modified mWHO OSI (17) was retrospectively calculated for each patient during their admission. The typical range for this validated measure is 0–8 (20–24). However, a “0” indicates no infection, and we did not enroll any subjects in this category. Furthermore, since we could not determine activity limitations for the enrolled subjects, we modified the criteria for “1” and “2”. A “1” in the original mWHO OSI represents “an infection, but ambulatory, with no activity limitation”. In our study, we defined a “1” as those seen in outpatient clinics and sent home. A “2” in the original mWHO OSI indicates “an infection, but ambulatory with activity limitations”. Our study defined a “2” as those seen in the ED and sent home. Those who scored a “3” were admitted to the hospital but did not require oxygen (O_2_), while those with a “4” were admitted to the hospital and required low-flow nasal cannula O_2_. Subjects scoring a “5” required a high-flow O_2_ by nasal cannula or noninvasive ventilation, and those scoring a “6” required endotracheal intubation and mechanical ventilation. A “7” on the mWHO OSI defines those mechanically ventilated patients receiving blood pressure support, RRT, or ECMO. An “8” represents a death that occurred during the study period, likely caused by the identified respiratory pathogen.

### Data validation

Manual validation of specific severity measures was conducted by CJ, ML, AD, and JK, focusing on patients admitted to the PICU. Key severity scale indicators were reviewed, including O_2_ flow (L/min), O_2_ flow categorization (high or low flow), non-invasive ventilation, mechanical ventilation, ECMO, pressor use, and RRT. If there were questions regarding diagnoses or other issues with the data, the final adjudication was performed by JK.

### Timing of NPIs

NPIs were implemented in Arkansas in late March 2020. These measures included universal masking, physical distancing, restrictions on public activities, and temporary closures of in-person schools and daycare facilities. Enhanced hand hygiene and surface cleaning were also promoted. NPIs were gradually lifted starting in March 2021. We used these periods to define the pre-NPI (January 2018 to March 2020), NPI (April 2020 to March 2021), and post-NPI (April 2021 to December 2024).

### Statistics

Descriptive statistics were used to summarize patient demographics, viral distributions, and clinical outcomes across the whole cohort and stratified by virus type, period (pre-NPI, NPI, post-NPI), rurality, and COI. Continuous variables, including age and mWHO OSI scores, were reported as means with standard deviations (SD). Categorical variables were summarized using frequencies and percentages.

Between-group comparisons of continuous variables (e.g., mWHO OSI scores by virus or time period) were assessed using one-way analysis of variance (ANOVA), Kruskal–Wallis, or Wilcoxon rank-sum tests, depending on data normality, with *post hoc* pairwise comparisons conducted using Dunn's test with Bonferroni correction. The Shapiro–Wilk test was employed to assess normality. Differences in categorical variables were evaluated using chi-square tests or Fisher's exact tests when appropriate. Temporal trends in severity were evaluated using non-parametric trend tests or linear regression models.

For stratified analyses of disease severity by RUCA category and COI category, a power analysis was performed to ensure sufficient sample size for virus-specific comparisons. Based on conventional thresholds and means [SD] similar to the analysis we first found between rural and urban populations (α = 0.05, power = 0.80), a minimum of 68 patients per RUCA group was required to detect an effect size in RUCA-based analyses. A minimum of 255 patients per group for COI-based analyses was required. Only viruses meeting these sample size thresholds in both comparison groups were included in severity analyses stratified by rurality or socioeconomic opportunity. All statistical tests were two-sided with a significance level of 0.05. Analyses were carried out using Microsoft Excel 365 (Microsoft Corp., Redmond, WA, USA) and R Studio (2025.05.0 + 496).

## Results

### Patient characteristics

Before exclusions were applied, we identified 92,595 children with a positive viral test from January 2018 to December 2024. There were 67,379 excluded because they did not have a diagnosis of asthma, and 15,278 were excluded either because the subjects were too young (<4 years), too old (>18 years), not from Arkansas, or had a positive bacterial infection identified on the RPP. After exclusions, we identified 9,391 pediatric patients with laboratory-confirmed respiratory viral infections who were also diagnosed with asthma during our specified time frame (Supplementary Figure S1). For these children, 78.8% (*n* = 7,400) had testing with the RPP, while 21.2% (*n* = 1,991) had testing with the 4-plex.

Table 1 highlights the total population demographics and the differences by virus among the population. As expected for the age group of children with asthma (18), most patients were male (58%) across all viral categories. The average age varied across virus groups, ranging from 7.91 years (SD 3.84) in the coinfections group to 10.5 years (SD 4.44) in those with SARS-CoV-2. The age distribution differed by virus, with RSV infections being most common among children aged 4–6 years (64.7%), while SARS-CoV-2 infections were more frequent in adolescents aged 13–18 years (37.2%). Race and ethnicity distributions showed virus-specific patterns: patients with INF A and B were disproportionately Black or African American (43% and 43.3%, respectively), whereas COV and PIV infections occurred more frequently in White children (56.8% and 52.9%, respectively). ADV and RSV were most prevalent in children of Hispanic ethnicity (26.3% and 23.6%, respectively). These variations underscore virus-specific demographic patterns that may reflect differences in susceptibility, exposure, healthcare access, or testing practices.

**Table 1:** Demographics of pediatric asthma subjects by virus.

Variables	*n* = 9,391	ADV (*n* = 297)	COV (*n* = 301)	HMPV (*n* = 402)	RV/EV (*n* = 3,535)	INF A (*n* = 1,194)	INF B (*n* = 180)	PIV (*n* = 480)	RSV (*n* = 861)	SARS-CoV-2 (*n* = 930)	Co-infections (*n* = 1,211)	*p*-value
Age, years (mean [SD])	7.91 [3.84]	7.46 [3.38]	8.54 [4.32]	7.15 [3.47]	7.77 [3.63]	9.04 [3.97]	8.71 [3.67]	7.40 [3.67]	6.70 [3.32]	10.5 [4.44]	7.91 [3.84]	*p* < 0.0001^a^
Female Sex, *n* (%)	3,944 (42%)	286 (40.5%)	219 (41.6%)	228 (40.5%)	1,838 (41.6%)	555 (41.4%)	87 (43.3%)	319 (42.2%)	474 (43.7%)	451 (41.6%)	493 (40.7%)	*p* < 0.0001^b^
Race, *n* (%)												
Nonhispanic White	4,115 (43.8%)	146 (49.2%)	171 (56.8%)	211 (52.5%)	1,681 (47.6%)	357 (29.9%)	62 (34.4%)	254 (52.9%)	345 (40.1%)	308 (33.1%)	580 (47.9%)	*p* < 0.0001^b^
Nonhispanic Black or African American	2,253 (24%)	42 (14.1%)	37 (12.3%)	68 (16.9%)	662 (18.7%)	513 (43%)	78 (43.3%)	62 (12.9%)	214 (24.9%)	384 (41.3%)	193 (15.9%)	*p* < 0.0001^b^
American Indian or Alaska Native	31 (0.3%)	2 (0.7%)	0	2 (0.5%)	10 (0.3%)	2 (0.2%)	0	4 (0.8%)	0	3 (0.3%)	6 (0.5%)	*p* = 0.1,567
Native Hawaiian or Other Pacific Islander	209 (2.2%)	5 (1.7%)	2 (0.7%)	3 (0.7%)	80 (2.3%)	21 (1.8%)	11 (6.1%)	10 (2.1%)	20 (2.3%)	7 (0.8%)	50 (4.1%)	*p* = 0.0001
Asian	93 (1%)	4 (1.3%)	0	2 (0.5%)	40 (1.1%)	11 (0.9%)	2 (1.1%)	4 (0.8%)	9 (1%)	8 (0.9%)	13 (1.1%)	*p* = 0.7026
Multiracial	290 (3.1%)	5 (1.7%)	8 (2.7%)	13 (3.2%)	102 (2.9%)	37 (3.1%)	6 (3.3%)	26 (5.4%)	31 (3.6%)	22 (2.4%)	40 (3.3%)	*p* = 0.0971
Other	275 (2.9%)	11 (3.7%)	10 (3.3%)	17 (4.2%)	114 (3.2%)	31 (2.6%)	2 (1.1%)	16 (3.3%)	19.2 (2.2%)	20 (2.2%)	35 (2.9%)	*p* = 0.2425
Unknown/ Decline to Answer	92 (1%)	3 (1%)	2 (0.7%)	3 (0.7%)	42 (1.2%)	10 (0.8%)	0	6 (1.2%)	7 (0.8%)	6 (0.6%)	13 (1.1%)	*p* = 0.6651
Ethnicity, *n* (%)												
Not Hispanic or Latino/a	7,172 (76.4%)	501 (71%)	397 (75.5%)	433 (76.9%)	3,320 (75.2%)	1,061 (79.2%)	177 (88.1%)	580 (76.7%)	847 (74.9%)	854 (78.7%)	906 (74.8%)	*p* < 0.0001^b^
Hispanic or Latino/a	2,033 (21.6%)	186 (26.3%)	120 (22.8%)	118 (21%)	1,011 (22.9%)	249 (18.6%)	23 (11.4%)	161 (21.3%)	267 (23.6%)	204 (18.8%)	281 (23.3%)	*p* < 0.0001^b^
Other	160 (1.7%)	19 (2.7%)	7 (1.3%)	9 (1.6%)	70 (1.6%)	24 (1.8%)	1 (0.5%)	14 (1.9%)	16 (1.4%)	22 (2%)	20 (1.7%)	*p* = 0.0004
Unknown/ Decline to Answer	26 (0.3%)	0	2 (0.4%)	3 (0.5%)	13 (0.3%)	5 (0.4%)	0	1 (0.1%)	1 (0.1%)	5 (0.5%)	4 (0.3%)	*p* = 0.5707

a ANOVA.

b Chi-Square.

### Patient characteristics by study period (pre-NPI, NPI, and post-NPI)

Among the patients included in the study, differences in demographic characteristics were observed across the pre-NPI, NPI, and post-NPI periods. The age differed, with a higher average during the pre-NPI and NPI periods (mean [SD]; 8.1 [3.9] and 8.3 [3.9], respectively) compared to the post-NPI period (7.9 [3.8]). Race and ethnicity distributions varied over time. The proportion of White patients decreased from 59.9% pre-NPI to 45% during NPI and then to 41.9% post-NPI (*p* < 0.0001). Black or African American patients represented 24% of the overall population and decreased from pre-NPI; 21.6% to NPI; 20.4%, followed by an increase during the post-NPI (24.5%) period. Native Hawaiian or Other Pacific Islander patients were mainly represented in the post-NPI period (*n* = 187, 2.4%) compared to the pre-NPI and NPI periods (*n* = 5, 0.5% and *n* = 17, 3.2%, respectively). The proportion of Hispanic or Latino/a patients increased from 13.4% before the NPI to 25.6% during the NPI, before declining to 22.3% after the NPI (Supplementary Table S1).

### Frequency of viruses in children with asthma

**Figure 1:**
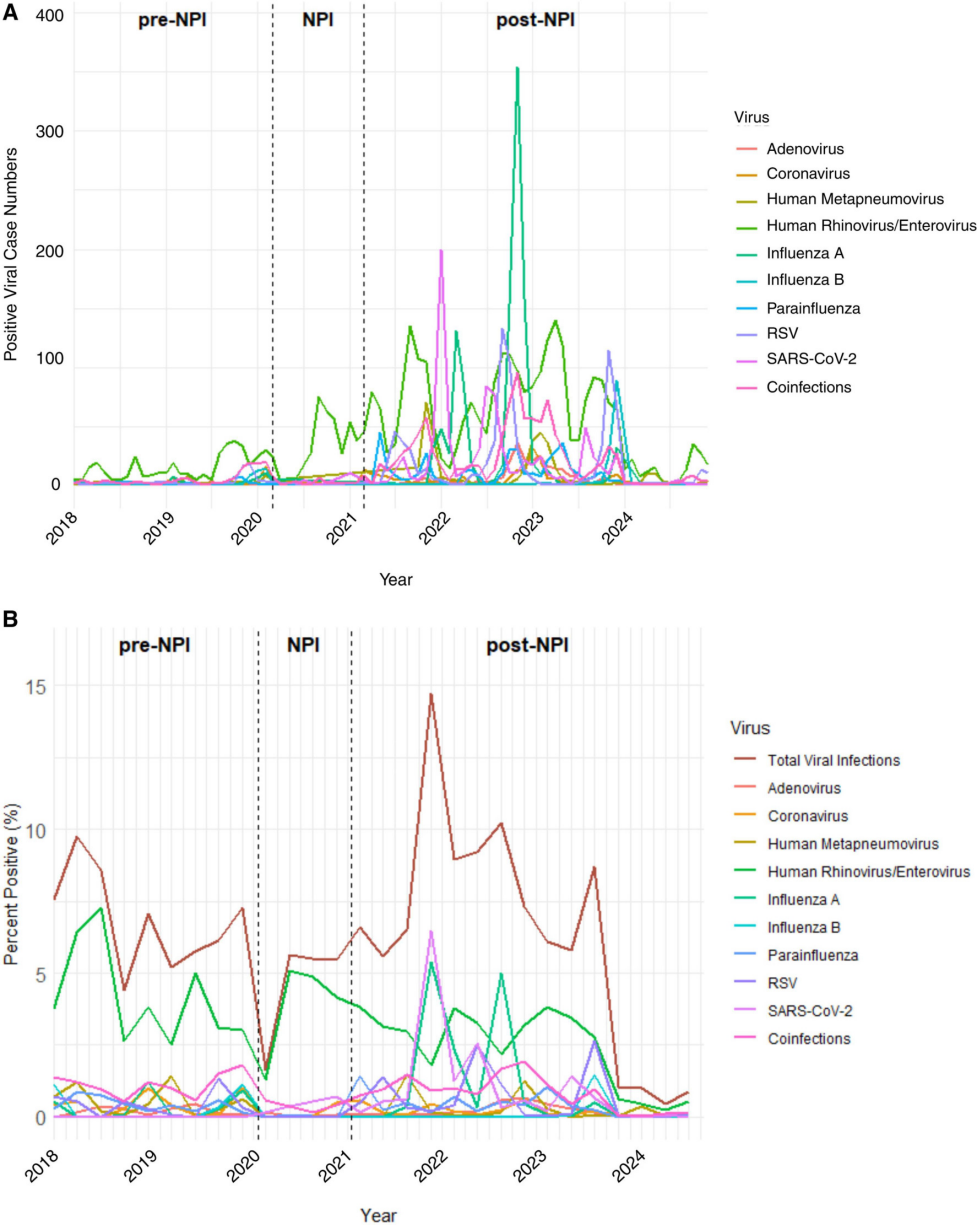
Monthly incidence and percent positivity of laboratory-confirmed respiratory viral infections in children with asthma from 2018 to 2024, stratified by virus type and public health intervention period*.* **(A)** This line graph depicts the number of children with asthma testing positive for respiratory viruses from January 2018 through December 2024. Each line represents a distinct viral pathogen: RSV, ADV, PIV, CoV (non-SARS-CoV-2), HMPV, INF A, RV/EV, SARS-CoV-2, INF B, and cases with multiple viral infections. The study period is divided into three intervals: pre-NPI, NPI, and post-NPI. **(B)** The percentage of children with asthma who tested positive for each virus per yearly quarters is shown to account for differences in testing volume over time. Percent positivity was calculated as the number of positive detections for each virus divided by the total number of tests performed that month. Peaks in percent positivity vary by virus and time, with some viruses (RV/EV, INF A, and coinfections) demonstrating distinct seasonal peaks. Vertical dashed lines denote the start and end of the NPI period.

The most common virus found in children with asthma during all periods was RV/EV, with 3,535 cases. INF A (*n* = 1,194), coinfections (*n* = 1,211), SARS-CoV-2 (*n* = 930), and RSV (*n* = 861) were also frequently observed. There were some variations in viral incidence based on the timing of NPIs for the types of respiratory viruses. Before the implementation of NPIs, seasonal peaks of RV/EV predominated, while the incidence of other viruses remained relatively low. During the NPI period, case numbers for nearly all viruses dropped dramatically, consistent with the impact of mitigation strategies, including masking, school closures, and physical distancing. Following the relaxation of NPIs, a significant rebound in viral activity occurred, including an unusual surge of RSV in July 2021 (*n* = 46) and a sharp, unprecedented peak in INF A cases in November 2022 (*n* = 354). RV/EV and ADV continued to circulate throughout all periods with less suppression compared to other viruses (Figure 1A).

### Quarterly positivity rates of respiratory viruses over time

Quarterly positivity rates for respiratory viruses among children with asthma from 2018 through 2024 were assessed, highlighting temporal shifts across the pre-NPI, NPI and post-NPI periods. A dramatic decline in viral detection occurred at the onset of the NPI period (Q1 2020). Positivity rates across nearly all viruses remained suppressed throughout the NPI period. Following the relaxation of NPIs, respiratory viruses re-emerged, with viral positivity rates for this cohort peaking in Q1 2022 (14.7%). Quarter 1 of 2022 also marked the highest detection of INF A (5.36%) and SARS-CoV-2 (6.45%), signaling overlapping seasonal surges. Coinfections and ADV peaked in Q1 2023 (1.91% and 0.68%, respectively), while RSV (2.63%) and INF B (1.46%) reached their highest detection in Q4 2023. Notably, RV/EV remained the most consistently detected virus, with a peak in the pre-NPI period (Q3 2018) but remaining an overall viral burden during both NPI and post-NPI periods. Several viruses showed relatively modest peak positivity rates, such as PIV (1.41% in Q2 2022) and HMPV (1.46% in Q4 2021). COV peaked in the pre-NPI period in Q1 2020 (1.02%) and was minimally detected afterward in this cohort. The quarterly positivity rate for all viruses drastically dropped in those diagnosed with asthma for quarters Q4 of 2023 (8.71%) to Q1 of 2024 (1.04%) (Figure 1B).

### Overall severity of illness in children with asthma and viral infections

**Figure 2:**
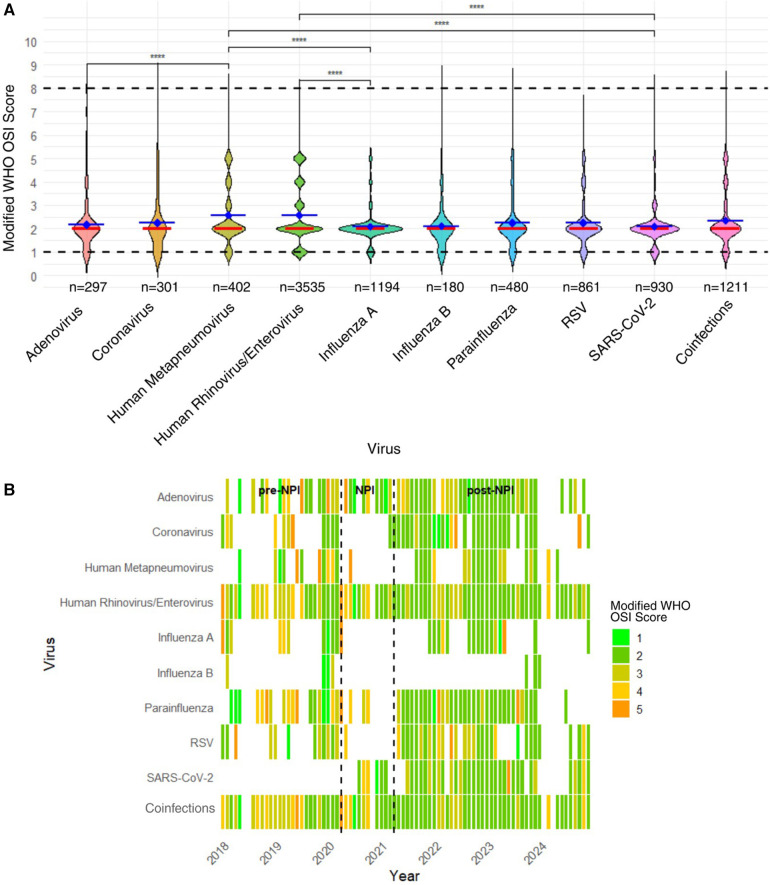
Modified mWHO OSI scores by viral pathogen among pediatric asthma patients, 2018–2024. **(A)** Mean mWHO OSI Scores by Viral Pathogens. This violin plot shows the mean (blue horizontal line) mWHO OSI scores (±SD) for children with asthma and laboratory-confirmed respiratory viral infections (*n* = 9,391), categorized by virus type with median lines in orange. Dashed horizontal lines indicate the measured mWHO OSI scores for our data set. Data shown outside of the dashed horizontal lines indicate the Kernel density estimation of the violin plot. Scores ranged from approximately 2.2 to 2.8, indicating a generally mild to moderate severity of illness. **(B)** Monthly mean mWHO OSI severity scores by viral pathogen from 2018 to 2024 among pediatric asthma patients. This heatmap displays the monthly average mWHO OSI scores by virus type in children with asthma over six years. Each tile represents the mean severity score for a given virus in each month, with colors ranging from green (mild illness, score = 1) to dark orange (more severe illness, score = 5). While most infections across viruses remained in the mild-to-moderate range, fluctuations in severity were noted over time, with higher mean severity scores observed intermittently, particularly for Parainfluenza, Influenza A, and coinfections. These temporal shifts highlight the dynamic nature of respiratory viral severity and the potential modifying impact of public health interventions on clinical outcomes.

We assessed the average mWHO OSI scores across various respiratory viruses in pediatric patients for the entire study period (2018–2024). Mean severity scores were generally low across all viruses, ranging from 2.09 [0.71] for INF A to 2.58 [1.31] for RV/EV. Infections with HMPV (2.58 [1.31]) and RV/EV exhibited the highest average mWHO OSI scores. In contrast, ADV (2.18 [1.05]), INF A (2.09 [0.71]), INF B (2.11 [1.02]), RSV (2.25 [1.01]), and SARS-CoV-2 (2.09 [0.82]) all displayed lower severity scores. Although confidence intervals overlapped across most viral groups, certain pairwise comparisons indicated differences. For example, RV/EV showed higher severity scores compared to INF A (*p* < 0.0001). HMPV severity was greater than that of ADV, INF A, and SARS-CoV-2 (*p* < 0.0001). SARS-CoV-2 had lower severity scores than several infections and RV/EV (*p* < 0.0001) (Figure 2A).

When examining monthly trends in mean mWHO OSI scores by virus, most infections across viral types and monthly time points were associated with mild (scores 1–2, green to dark green) to moderate (scores 3–5, yellow to orange) severity. RV/EV, the most frequently detected virus, consistently exhibited mild average severity, particularly in the post-NPI period. INF A, coinfections, and RSV exhibited more variability, with intermittent periods of moderate illness occurring before and shortly after the NPI period. Winter months often correspond to elevated severity scores across several viruses. The year 2019 exhibited several isolated months with elevated severity (scores ≥3), especially those subjects with RSV, INF A, and COV. More severe average scores (≥4) were rare and appeared sporadically, most notably with PIV, HMPV, and RSV in isolated pre-NPI or early post-NPI periods. Overall, the heatmap highlights the temporal and pathogen-specific variability in illness severity but underscores that sustained high-severity disease was uncommon across the study period (Figure 2B).

### Temporal trends in mWHO OSI scores by virus in children with asthma

**Figure 3:**
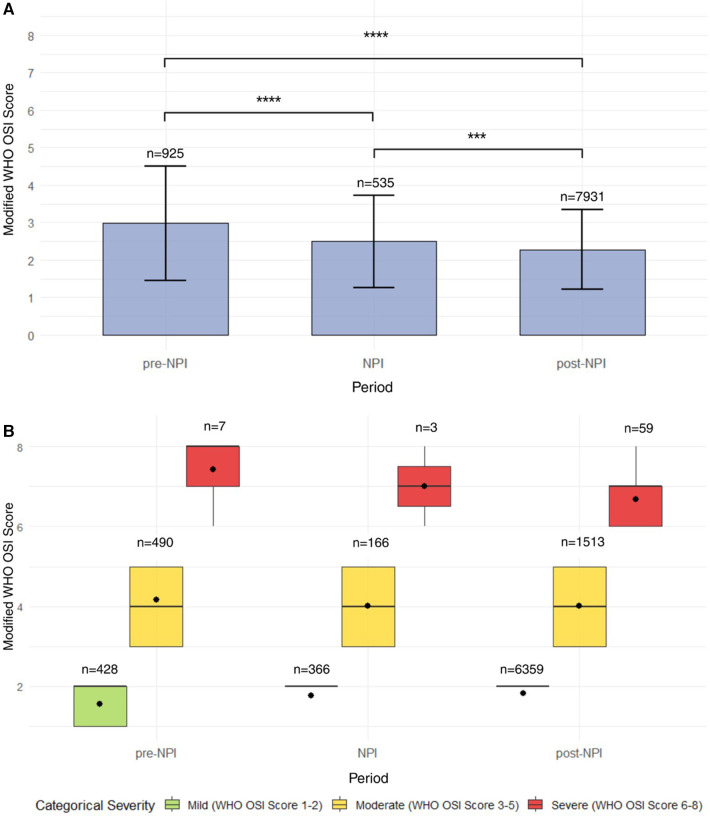
Distribution and severity trends of mWHO OSI scores across NPI periods in pediatric asthma patients with viral infections. **(A)** Distribution of mWHO OSI scores across pre-NPI, NPI, and post-NPI periods in pediatric asthma patients with respiratory viral infections. This bar plot illustrates the distribution of mWHO OSI scores by public health intervention period: pre-NPI, NPI, and post-NPI. Mean severity scores and standard deviations are shown for each period. Illness severity was highest in the pre-NPI period, declined during the NPI period, and was lowest in the post-NPI period, with minimal variability and a near-uniform score of 2. **(B)** mWHO OSI scores stratified by severity category and time period in pediatric asthma patients with viral infections. This boxplot displays mWHO OSI scores categorized as mild (green), moderate (yellow), or severe (red) across three public health periods: pre-NPI, NPI, and post-NPI. While the majority of cases in all periods were mild to moderate, severe cases (score ≥6) were rare but present in each time frame, with the largest cluster observed post-NPI (*n* = 59). Black dots represent mean scores for each group. These data suggest a sustained reduction in illness severity over time, though a small number of severe outcomes persisted even after the relaxation of pandemic-related interventions.

Next, we examined the distribution of mWHO OSI scores across three time periods, pre-NPI, NPI, and post-NPI, highlighting changes in disease severity with the implementation of these guidelines. During the pre-NPI period (*n* = 925), the mean mWHO OSI score was 2.98 [1.53]. In the NPI period (*n* = 535), the mean severity declined to 2.49 [1.24], and the standard deviation narrowed, suggesting more uniform and less severe outcomes during this time. By the post-NPI period (*n* = 7,931, mWHO OSI scores were consistently low, with a tight interquartile range, reflecting a reduction in disease severity across the children with asthma in this population (2.28 [1.06]) (Figure 3A).

Next, we evaluated mWHO OSI score distributions by categorical severity level (mild, moderate, and severe) as above across the three time periods. During the pre-NPI period, moderate severity cases predominated (*n* = 490; 4.16 [0.84]), with fewer mild cases (*n* = 428; 1.55 [0.50]) and very few severe cases (*n* = 7; 7.43 [0.98]). During the NPI period, fewer children with asthma were moderate in severity (*n* = 166; 4.01 [0.83]). Mild cases also slightly declined (*n* = 366; 1.77 [0.42]), and severe cases remained rare (*n* = 3; 7.00 [1.00]). In the post-NPI period, there was a notable increase in mild cases (*n* = 6,359; 1.82 [0.38]) and in moderate cases (*n* = 1,513; 4.02 [0.80]). There was also a modest increase in severe cases (*n* = 59; 6.68 [0.65]), although severe cases still represented a small fraction overall. Across all periods, most pediatric patients presented with either mild or moderate illness, and severe disease remained uncommon (Figure 3B).

To further characterize severity trends, viral pathogens were stratified by NPI period and mWHO OSI categorical severity. Across all viruses, the majority of cases occurred in the post-NPI period and were classified as mild. RV/EV accounted for the highest total case count (*n* = 3,535), with most cases occurring post-NPI (*n* = 2,683) and presenting as mild (*n* = 1,877). INF A (*n* = 1,194), SARS-CoV-2 (*n* = 930), RSV (*n* = 861), and coinfections (*n* = 1,211) were also primarily observed post-NPI, with distributions skewed toward mild illness. Severe cases were rare across all viruses and time periods (Supplementary Figure S2).

The implementation and relaxation of NPIs were also associated with significant changes in secondary clinical outcomes among pediatric patients with respiratory infections. In our population, the number of children with asthma seen in the ED and sent home increased across periods, from 71.2% pre-NPI to 84.3% post-NPI (*p* < 0.0001). In contrast, markers of disease severity—including hospital admissions (53.7% to 19.8%), PICU admissions (14.3% to 3.1%), low- and high-flow O_2_ support (16.2% and 22.7% to 7.5% and 6.6%, respectively), non-invasive ventilation (6.2% to 1.1%), and pressor use (5.5% to 1.6%)—all declined over time (*p* < 0.0001). Mechanical ventilation use was low across all periods, while ECMO use and mortality were rare, showing borderline changes or no differences over the periods (*p* = 0.004 and *p* = 0.01, respectively) (Table 2).

**Table 2:** Secondary measures of severity by NPI period.

Variables	*n* = 9,391	pre-NPI (*n* = 925)	NPI (*n* = 535)	post-NPI (*n* = 7,931)	*p*-value
ED Visit, *n* (%)	7,782 (82.9%)	659 (71.2%)	441 (82.4%)	6,682 (84.3%)	*p* < 0.0001^a^
Hospital Admission, *n* (%)	2,238 (23.8%)	497 (53.7%)	169 (31.6%)	1,572 (19.8%)	*p* < 0.0001^a^
PICU Admission, *n* (%)	416 (4.4%)	132 (14.3%)	38 (7.1%)	246 (3.1%)	*p* < 0.0001^a^
O2 Flow (Low), *n* (%)	800 (8.5%)	150 (16.2%)	55 (10.3%)	595 (7.5%)	*p* < 0.0001^a^
O2 Flow (High), *n* (%)	795 (8.5%)	210 (22.7%)	58 (10.8%)	527 (6.6%)	*p* < 0.0001^a^
Non-invasive Ventilation, *n* (%)	156 (1.7%)	57 (6.2%)	14 (2.6%)	85 (1.1%)	*p* < 0.0001^a^
Mechanical Ventilation, *n* (%)	61 (0.6%)	3 (0.3%)	2 (0.4%)	56 (0.7%)	*p* < 0.0001^a^
ECMO, *n* (%)	14 (0.1%)	4 (0.4%)	0 (0.0%)	10 (0.1%)	*p* = 0.004^b^
Pressor Support, *n* (%)	198 (2.1%)	56 (5.5%)	23 (4.3%)	124 (1.6%)	*p* < 0.0001^a^
Mortality, *n* (%)	12 (0.1%)	5 (0.5%)	1 (0.2%)	6 (0.1%)	*p* < 0.0001^b^

a Chi-Square.

b Fisher's Exact.

### Longitudinal changes in mWHO OSI scores by virus in children with asthma

We assessed the mean mWHO OSI scores over time for asthma patients infected with each of the respiratory viruses tested. During the pre-NPI period, most viruses were associated with moderate severity (mean scores between 2.5 and 3.5), with RV/EV (mean [SD]; 3.19 [1.56]), HMPV (3.23 [1.64]), ADV (3.05 [1.79]), and coinfections (2.59 [1.43]) showing slightly higher scores. The NPI period had considerably smaller sample sizes; however, mean scores remained generally stable. In the post-NPI period, a decline in mean mWHO OSI scores was observed across nearly all viral groups, with scores clustering closer to 2, indicating milder clinical courses during this time. Notably, SARS-CoV-2 exhibited relatively consistent severity across the NPI (2.22 [0.88]) and post-NPI (2.08 [0.81]) periods, while Co-infections did not show greater severity than single-virus infections across all periods. Overall, the post-NPI era was associated with reduced disease severity and less variability in clinical outcomes (Supplementary Figure S3).

### Differences in the severity of illness for respiratory infections in children with asthma by rural/urban status

Across all viral infections, most cases occurred in children considered urban. Rural cases made up a small proportion of the total cases per virus. RV/EV was the most common virus in both rural (*n* = 285) and urban (*n* = 3,250) populations. Although there are slight variations, no virus showed a strong preference for rural over urban populations. Overall, the severity of illness was higher in rural populations (*n* = 611) compared to urban populations (*n* = 8,780) (mean [SD]; 3.01 [1.47], 2.31 [1.11], respectively; *p* < 0.0001) for the entire study.

**Figure 4:**
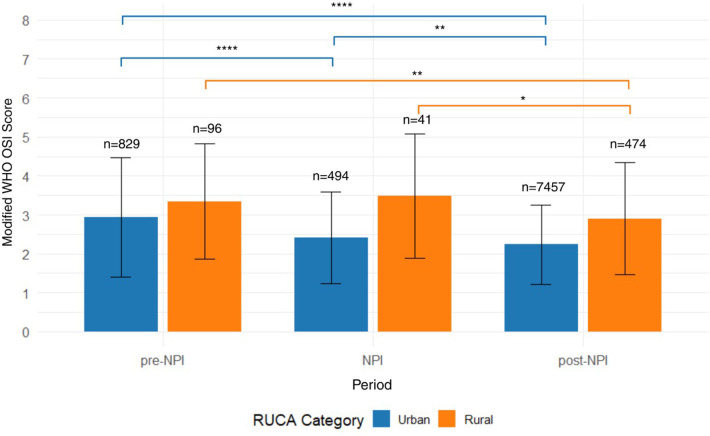
Comparison of mWHO OSI scores by rurality across public health intervention periods in pediatric asthma patients with viral infections. This bar plot displays mWHO OSI scores stratified by RUCA-defined rural (orange) and urban (blue) residence across three time periods: pre-NPI, NPI, and post-NPI. Mean severity scores and standard deviations are shown for each RUCA category, with sample sizes indicated above the boxes. Across all periods, rural patients tended to exhibit slightly mean severity scores compared to urban patients, particularly during the NPI and post-NPI periods. These findings suggest persistent disparities in clinical severity of viral illness by geographic location throughout the COVID-19 pandemic and its aftermath.

We separated subjects by mWHO OSI scores and rural-urban classification (RUCA category) across three time periods. In the pre-NPI period, rural patients (*n* = 96) exhibited a higher mean mWHO OSI score (mean [SD], 3.34 [1.48]) and greater variability than urban patients (*n* = 829; 2.94 [1.53]), who showed lower and mean scores with a narrower distribution (*p* = 0.013). A similar pattern persisted during the NPI period, with rural patients (*n* = 41) again demonstrating higher mean severity (3.49 [1.60]) and wider standard deviations compared to their urban counterparts (*n* = 494; 2.41[1.17]). In the post-NPI period, most cases were urban (*n* = 7,457) and displayed relatively low and consistent mWHO OSI scores (2.24 [1.02]). Rural patients (*n* = 474) had slightly higher values (2.91 [1.44]; *p* < 0.0001) (Figure 4).

In rural patients, scores decreased from the pre-NPI (*n* = 96) to post-NPI (*n* = 474) periods (3.34 [1.48], 2.91 [1.44], respectively; *p* = 0.00315). The change from NPI (*n* = 41) to post-NPI trended toward a difference (3.49 [1.60], 2.91 [1.44], respectively; *p* = 0.0160), whereas comparing pre-NPI to NPI showed no differences (3.34 [1.48], 3.49 [1.60], respectively; *p* = 0.741). Urban patients demonstrated clear and consistent improvement in severity scores over time, with the largest reductions observed between the pre-NPI (*n* = 829) and post-NPI (*n* = 7,457) periods (2.94 [1.53], 2.24 [1.02], respectively; *p* < 0.0001). There was also a difference between the pre-NPI and NPI (*n* = 494) periods (2.94 [1.53], 2.41 [1.17], respectively; *p* < 0.0001) and between the NPI and post-NPI periods (2.41 [1.17], 2.24 [1.02], respectively; *p* = 0.001) (Figure 4).

To further explore the interaction between illness severity and viral pathogens, we stratified mWHO OSI scores by RUCA classification and virus type. A power analysis determined that a minimum of 68 subjects in both urban and rural groups was required for reliable comparisons; only RV/EV and coinfections met this threshold and were included. Across both viruses, rural patients exhibited higher mean severity scores than their urban counterparts. Among RV/EV cases, rural patients (*n* = 285) had a higher average mWHO OSI score (mean [SD], 3.25 [1.52] compared to urban patients (*n* = 3,250; 2.52 [1.28]). A similar trend was seen in coinfections, where rural cases (*n* = 94) had higher mean severity scores (2.94 [1.46]) than urban cases (*n* = 1,117; 2.30 [1.09]) (Supplementary Figure S4).

### Differences in illness severity for respiratory infections in children with asthma by childhood opportunity index

The distribution of respiratory viruses remained relatively consistent across COI categories. RV/EV and coinfections were among the most prevalent in all COI groups. No substantial differences in virus type prevalence were observed based on COI data. mWHO OSI scores differed by COI category over the whole study, with higher average scores observed in the very low/low group (*n* = 2,396) compared to the moderate group (*n* = 1,697) and the high/very high group (*n* = 5,298) (mean [SD]; 2.61 [1.32], 2.28 [1.04], 2.27 [1.08], respectively; *p* < 0.0001). Scores between the moderate and high/very high groups were not notably different (*p* = 0.7877).

**Figure 5:**
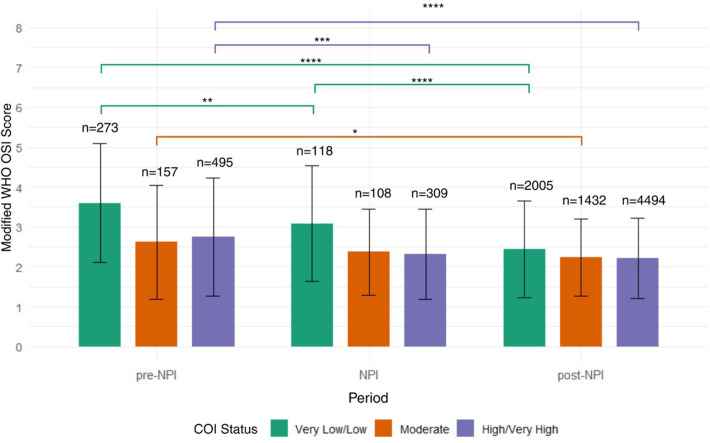
mWHO OSI scores Child Opportunity Index (COI) and NPI period in pediatric asthma patients with viral infections. This bar plot illustrates mWHO OSI scores across three time periods, pre-NPI, NPI, and post-NPI, stratified by COI categories: Very Low/Low (green), Moderate (orange), and High/Very High (purple). Mean severity scores and standard deviations are displayed. Prior to and during the NPI period, children in the High/Very High COI exhibited comparable or slightly higher mean and median severity scores relative to other groups. By the post-NPI period, severity scores declined across all COI levels, with reduced variability, suggesting a broad shift toward milder illness regardless of underlying social vulnerability.

Next, we stratified mWHO OSI score distributions by COI, categorizing the COI variables into very low/low, moderate, and high/very high COI across pre-NPI, NPI, and post-NPI. During the pre-NPI period, patients from very low/low opportunity areas (*n* = 273) exhibited the highest variability in severity scores (mean [SD]; 3.60 [1.49]) and a higher mean score compared to those from moderate (*n* = 157, mean [SD]; 2.62 [1.43]) and high/very high (*n* = 495; 2.76 [1.48]) opportunity areas. Both moderate and high/very high opportunity groups had lower severity scores than the very low/low group (*p* < 0.0001). During the NPI period, the mean severity scores decreased across all COI categories, particularly in the moderate (2.37 [1.08]) and high/very high (2.31 [1.13]) COI groups. Patients from the very low/low COI group had higher severity scores (3.08 [1.45]). In the post-NPI period, all groups displayed low and consistent severity scores, with minimal variability and means around 2.0 (very low/low 2.45 [1.21], moderate 2.23 [0.97], high/very high 2.21 [1.01]) (Figure 5).

To assess whether COI influenced severity across viral infections, we stratified mWHO OSI scores by COI category and virus type. A power analysis indicated that a minimum of 255 patients per COI group was required for inclusion. No viruses had sufficient sample size within the moderate COI group and were therefore excluded from this analysis. RV/EV, INF A, and SARS-CoV-2 met inclusion criteria for both the very low/low and high/very high COI categories and were analyzed. Among patients with RV/EV, those from very low/ low opportunity areas (*n* = 762) had higher mean severity scores (mean [SD], 3.17 [1.46]) compared to those from high/very high areas (*n* = 2,114; 2.41 [1.23]). This pattern was consistent across INF A, and SARS-CoV-2, although differences were more modest. For INF A, the very low/low group (*n* = 459) and high/very high group (*n* = 588) had similar mean scores (2.04 [0.72], 2.12 [0.73], respectively). For SARS-CoV-2, the scores were also similar (very low/low; 2.1 [0.89], high/very high; 2.08 [0.76]) (Supplementary Figure S5).

## Discussion

This study examined over 9,000 pediatric asthma patients with laboratory-confirmed respiratory viral infections from 2018 to 2024. We noted significant temporal, demographic, and clinical trends influenced by NPIs implemented during the COVID-19 pandemic. Our results indicate that public health measures had a notable impact on both the patterns of viral circulation and the severity of disease, with lasting decreases in severe outcomes in the post-NPI period.

The temporal distribution of viral infections was profoundly affected by NPIs. Consistent with national surveillance trends, we observed an abrupt decline in respiratory virus activity during the NPI period, followed by dramatic rebounds upon the relaxation of these public health measures. The unusual surge in RSV during late 2021 and the sharp peak of INF A in late 2022 illustrate the reshaping of seasonal transmission patterns in the post-NPI era. These trends align with international reports of disrupted respiratory virus seasonality following the removal of NPIs and support global shifts in viral transmission, with the hypothesis of “immunity debt” potentially explaining these atypical surges (19–21). RV/EV circulated consistently across all time points, demonstrating relative resilience to mitigation strategies, a finding also observed in other pediatric cohorts. This aligns with reports from pediatric and adult studies in Canada indicating that rhinoviruses are less susceptible to mitigation strategies due to their environmental stability and diverse transmission routes (22).

Despite the resurgence of respiratory viruses, the severity of disease seemingly decreased over time. During the pre-NPI phase, mWHO OSI scores were higher and more variable, indicating more severe disease presentations. In contrast, the post-NPI period was marked by a higher number of mild cases and a notably lower proportion of patients with moderate or severe illness. These results suggest a temporal trend toward milder clinical presentations following the implementation and eventual relaxation of NPIs. These trends were consistent across various viral types, including RV/EV, INF A, RSV, and SARS-CoV-2, and were supported by both continuous and categorical severity assessments. These findings raise questions about the “immunity debt” hypothesis, as one might expect worse illness in those who had no immunity to the viruses in question. However, it is also possible that these types of infections, or viral detections, were present before NPIs. These children with mild disease may not have been tested at that time because there is little that could be done for viral infections. With the advent of Paxlovid and other options for the treatment of SARS-CoV-2, more and more children are being tested for viruses. Future studies should include serum antibody testing for the viruses in question, along with measures of illness severity, which might help to resolve this issue.

These studies also suggest that increased testing occurred in patients with less severe symptoms. This aligns with data from a recent study by *Molloy* et al., which reported a 4.6-fold increase in respiratory pathogen testing among children and adolescents presenting to the ED or requiring hospitalization for respiratory illnesses between 2016 and 2023 (from 13.6% in 2016 to a peak of 62.2% in 2022). The most pronounced rise occurred in patients treated and discharged from the ED, where testing increased 6.7-fold (from 8.8% in 2016 to 59.3% in 2022) (23). However, the Molloy study did not assess illness severity, which limited its ability to determine whether increased testing had an impact on clinical outcomes. Our study suggests that the increase in viral testing has led to the detection of more viruses, but typically only in patients with less severe illness.

Although healthcare utilization increased over time in our study, the reduction in disease severity over time aligns with recent studies showing decreased pediatric hospitalization rates and a lower severity of SARS-CoV-2 during the Omicron variant wave (24). Only a small fraction of patients in our study ever met the criteria for severe illness (mWHO OSI 6–8), reinforcing the overall mildness of viral infections in this asthma cohort. A study utilizing secondary outcomes (asthma severity score, asthma diary, and cough diary scores) found that the presence of a viral respiratory illness had a modest effect on asthma severity (25). *Dinwiddie* et al. noted that asthma control and allergic sensitization influence the severity of virus-related asthma exacerbations in children presenting to the ED. Among 120 pediatric patients, those with uncontrolled asthma and viral infection, especially rhinovirus, had more severe symptoms, particularly when allergy was also present (26).

Secondary clinical outcomes followed a similar trajectory in our study. Although ED utilization increased over time, likely reflecting greater health-seeking behavior or broader testing availability, the incidence of severe clinical outcomes declined. Specifically, we observed reductions in hospital and PICU admissions, need for O_2_ support (both low- and high-flow), non-invasive ventilation, and vasopressor use during the post-NPI period. These findings align with a recent study by *Caid* et al., which reported a return to pre-NPI levels of asthma-related ED visits after public health measures were relaxed, without a corresponding increase in hospitalizations or ICU admissions (14). In our study, the use of mechanical ventilation, ECMO, and the incidence of mortality remained rare. Taken together, these results suggest a sustained trend toward less severe disease presentations over time, even as the frequency of healthcare encounters rose.

Patient demographics varied by viral etiology, highlighting potential differences in exposure risk, healthcare-seeking behavior, or host susceptibility. The median age differed across virus groups, with RSV primarily affecting younger children aged 4–6 years, while SARS-CoV-2 was more prevalent among adolescents with asthma. The predominance of male patients across all groups aligns with prior studies reporting higher asthma prevalence in boys during childhood (18). Notably, the distributions of race and ethnicity also varied by virus. Black or African American children were disproportionately represented among those infected with INF A and B, whereas White children were more frequently infected with COV and PIV. The burden of INF among Black and African American children aligns with CDC data showing lower INF vaccination coverage and higher hospitalization rates in this population (27, 28). These findings underscore a critical need for enhanced outreach and targeted vaccination campaigns to increase influenza vaccine uptake in underserved communities. However, our study did not obtain vaccine status for influenza, which limits our interpretation. Hispanic children represented the highest numbers among ADV and RSV cases. Given the recent guidelines for RSV vaccination in pregnant mothers, these findings could help to guide a directed therapeutic approach targeting those of Hispanic heritage. These demographic patterns highlight the intricate interplay of host, environmental, and structural factors in determining viral infection risk among children with asthma.

We observed disparities in the severity of illness based on rural/urban classification and COI. Throughout all time periods, children from rural areas exhibited higher and more variable mWHO OSI scores compared to their urban counterparts, suggesting persistent geographic disparities in disease severity or access to care. While this finding may seem to conflict with data from other studies on urban/rural asthma, which detail lower rates and severity of asthma in rural areas (29, 30), a previous study in our state showed higher rates of asthma prevalence and morbidity in rural areas (31). In this study, rural children with asthma had more recurrent trouble breathing (odds ratio [OR], 1.9; 95% confidence interval [CI], 1.5–2.2), recurrent cough (OR, 2.2; 95% CI, 1.9–2.6), recurrent chest tightness (OR, 1.8; 95% CI, 1.5–2.2), and repeated episodes of bronchitis (OR, 2.2; 95% CI, 1.7–2.8) during the preceding 2 years compared to urban children. These findings were present despite similar healthcare utilization between the groups (31).

Children from very low/low COI communities had higher severity scores in the pre-NPI period compared to those from moderate or high-opportunity areas. This suggests that children from more disadvantaged communities may have experienced more severe disease before the implementation of NPIs. This aligns with previous research demonstrating that children from low COI neighborhoods are more likely to experience exacerbation-prone asthma and increased emergency care utilization (32). Overall, the data suggest that community-level socioeconomic factors may have had a more significant impact on clinical severity before the pandemic. However, these differences diminished over time, with all COI groups showing lower severity scores by the post-NPI era. These findings suggest that while structural and geographic disparities influenced outcomes early in the study period, the overall decline in disease severity may have helped lessen these differences in later years.

The limitations of this study include potential changes in testing practices and healthcare access over time, especially during the COVID-19 pandemic, which may have influenced case detection and outcome ascertainment. Additionally, while the mWHO OSI scale provides a standardized measure of clinical severity, its application in pediatric populations with asthma may not fully capture the nuances of disease presentation. Our analysis is restricted to patients from Arkansas, which might affect the generalizability to other geographic regions. Finally, our study was retrospective, leaving many unmeasured confounding variables, including environmental exposures, housing conditions, and family-level socioeconomic status, that could influence disease severity. We were unable to obtain complete demographic data for patients who tested negative for viral infections, which skews the total and specific viral positivity rates lower than they should be as our asthma population is 4–18 with an ICD-10 code positive detection for an asthma diagnosis. Another key limitation was statistical power in stratified analysis. While our total sample size was robust, power analyses required minimum group sizes to assess severity by virus type. Many viruses did not meet these thresholds in rural or very low/low opportunity groups, limiting our ability to evaluate geographic and socioeconomic effects across all viral infections. As a result, findings from stratified analyses are limited to a subset of viruses with sufficient sample size and may not generalize to all pathogens of interest.

In conclusion, our findings emphasize the impact of NPIs on viral transmission and clinical severity in children with asthma. The post-NPI era was characterized by a notable decline in severe illness, despite rising viral circulation and increased healthcare utilization. Ongoing surveillance is essential to determine whether these trends continue and to monitor the potential re-emergence of disparities in respiratory outcomes across demographic and geographic lines.

## Data Availability

The raw data supporting the conclusions of this article will be made available by the authors, without undue reservation.
