# Supplementary material for: From lockdown to recovery: changing patterns of viral infection severity in a pediatric cohort with asthma
Source: Front Allergy. 2025 Sep 24;6:1645968. doi: 10.3389/falgy.2025.1645968 (PMC12504475; doi:10.3389/falgy.2025.1645968)
Supplement: Supplementary file 1 [file Table1.docx]

|  | **n=9,391** | **pre-NPI (n=925)** | **NPI (n=535)** | **post-NPI (n=7391)** | **p-value** |
| --- | --- | --- | --- | --- | --- |
| **Age (mean [SD])** | 7.9 [3.8] | 8.1 [3.9] | 8.3 [3.9] | 7.9 [3.8] | 0.007* |
| **Female Sex, n (%)** | 3944 (42%) | 416 (45%) | 225 (42.1%) | 3303 (41.6%) | 0.152# |
| **Race, n (%)** |  |  |  |  |  |
| **Non-Hispanic White** | 4115 (43.8%) | 554 (59.9%) | 241 (45%) | 3320 (41.9%) | <0.0001# |
| **Non-Hispanic Black or African American** | 2253 (24%) | 200 (21.6%) | 109 (20.4%) | 1944 (24.5%) | 0.02# |
| **American Indian or Alaska Native** | 31 (0.3%) | 0 (0%) | 0 (0%) | 31 (0.4%) | 0.056~ |
| **Native Hawaiian or Other Pacific Islander** | 209 (2.2%) | 5 (0.5%) | 17 (3.2%) | 187 (2.4%) | <0.0001# |
| **Asian** | 93 (1%) | 4 (0.4%) | 4 (0.7%) | 85 (1.1%) | 0.154~ |
| **Multiracial** | 290 (3.1%) | 21 (2.3%) | 15 (2.8%) | 254 (3.2%) | 0.278# |
| **Other** | 275 (2.9%) | 13 (1.4%) | 10 (1.9%) | 252 (3.2%) | 0.003# |
| **Unknown/ Decline to Answer** | 92 (1%) | 4 (0.4%) | 2 (0.4%) | 86 (1.1%) | 0.059~ |
| **Ethnicity, n (%)** |  |  |  |  |  |
| **Not Hispanic or Latino/a** | 7172 (76.4%) | 793 (85.7%) | 393 (73.5%) | 5986 (75.5%) | <0.0001# |
| **Hispanic or Latino/a** | 2033 (21.6%) | 124 (13.4%) | 137 (25.6%) | 1772 (22.3%) | <0.0001# |
| **Other** | 160 (1.7%) | 6 (0.6%) | 4 (0.7%) | 150 (1.9%) | 0.003~ |
| **Unknown/ Decline to Answer** | 26 (0.3%) | 2 (0.2%) | 1 (0.2%) | 23 (0.3%) | 1~ |
| **Virus, n (%)** |  |  |  |  |  |
| **ADV** | 297 (3.6%) | 22 (2.8%) | 6 (1.2%) | 269 (3.9%) | 0.004# |
| **COV** | 301 (3.7%) | 54 (7%) | 18 (3.6%) | 229 (3.3%) | <0.0001# |
| **HMPV** | 402 (4.9%) | 56 (7.2%) | 0 (0%) | 346 (5%) | <0.0001~ |
| **RV/EV** | 3535 (43.2%) | 432 (55.7%) | 420 (84%) | 2683 (38.9%) | <0.0001# |
| **INF A** | 1194 (14.6%) | 51 (6.6%) | 0 (0%) | 1143 (16.6%) | <0.0001~ |
| **INF B** | 180 (2.2%) | 48 (6.2%) | 1 (0.2%) | 131 (1.9%) | <0.0001~ |
| **PIV** | 480 (5.9%) | 46 (5.9%) | 3 (0.6%) | 431 (6.2%) | <0.0001~ |
| **RSV** | 861 (10.5%) | 66 (8.5%) | 1 (0.2%) | 794 (11.5%) | <0.0001~ |
| **SARS-CoV-2** | 930 (11.4%) | 0 (0%) | 51 (10.2%) | 879 (12.7%) | <0.0001~ |
| **Co-Infections** | 1211 (12.9%) | 150 (16.2%) | 35 (6.5%) | 1026 (12.9%) | <0.0001# |
| ***ANOVA** |  |  |  |  |  |
| **#Chi-Squared** |  |  |  |  |  |
| **~Fisher's Exact** |  |  |  |  |  |

**Supplemental Table 1. Demographics by NPI period.**
