# Supplementary material for: From lockdown to recovery: changing patterns of viral infection severity in a pediatric cohort with asthma
Source: Front Allergy. 2025 Sep 24;6:1645968. doi: 10.3389/falgy.2025.1645968 (PMC12504475; doi:10.3389/falgy.2025.1645968)

**Supplemental Figure 1.**





**Supplemental Figure 2.**


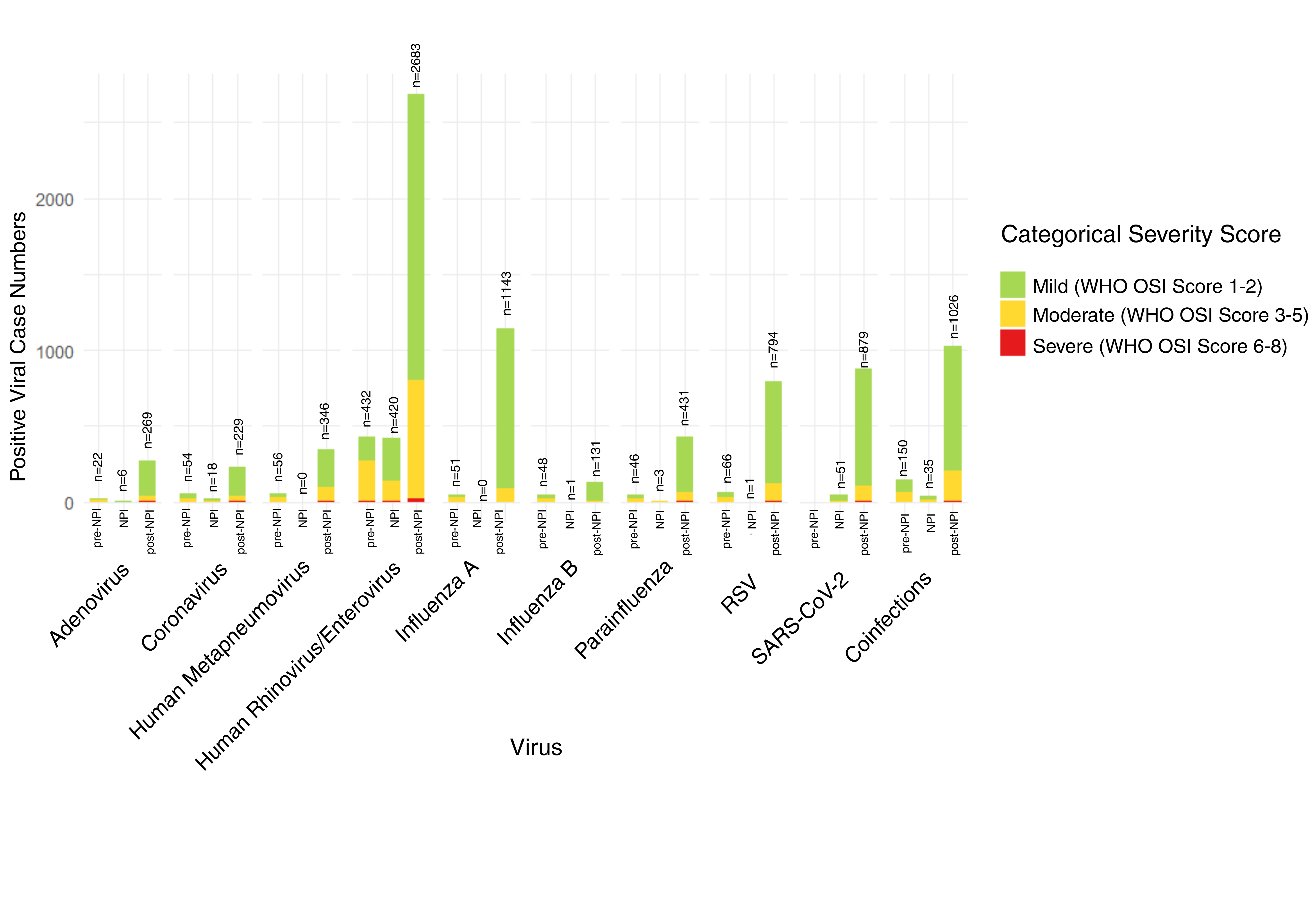


**Supplemental Figure 3.**
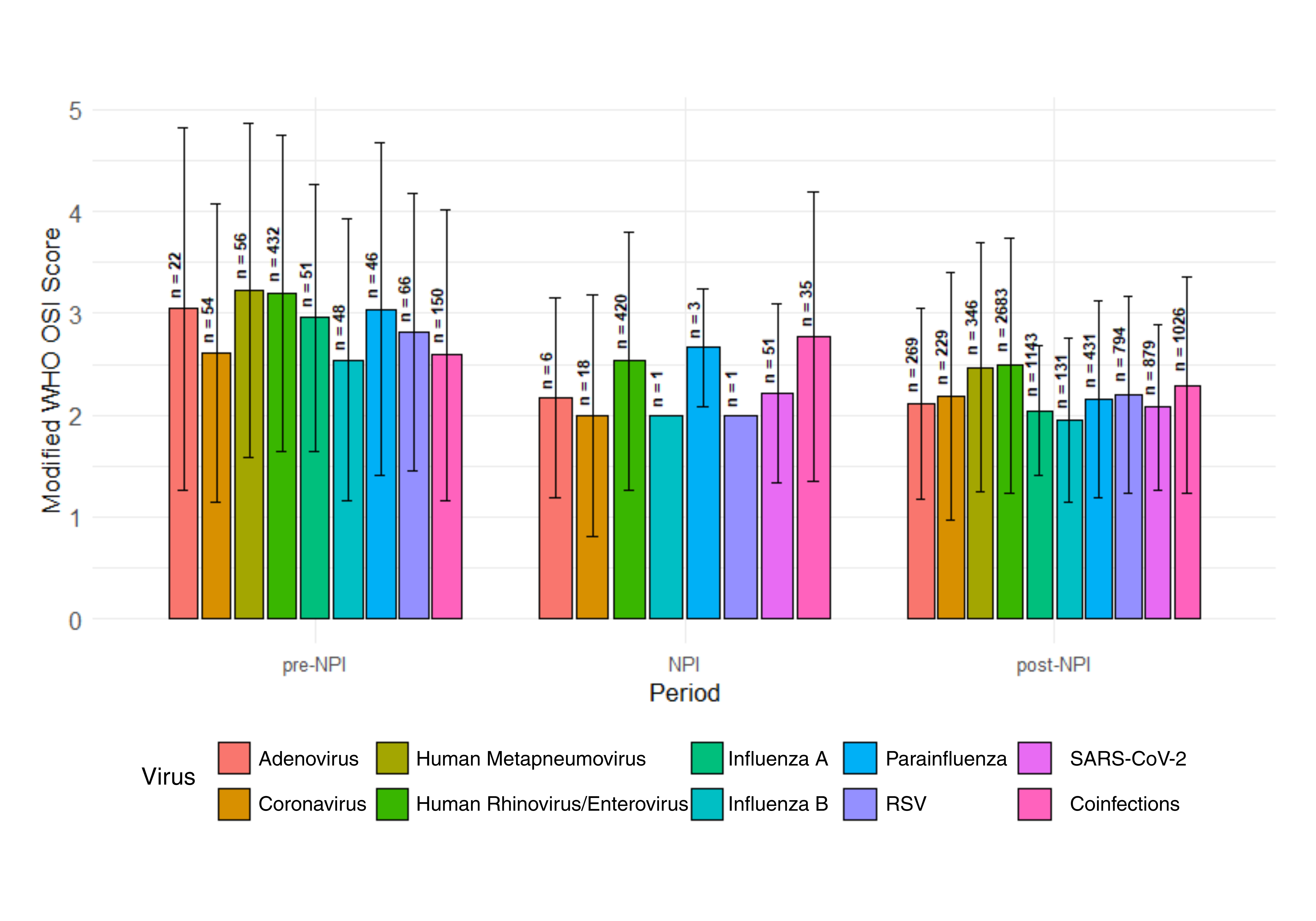


**Supplemental Figure 4.**
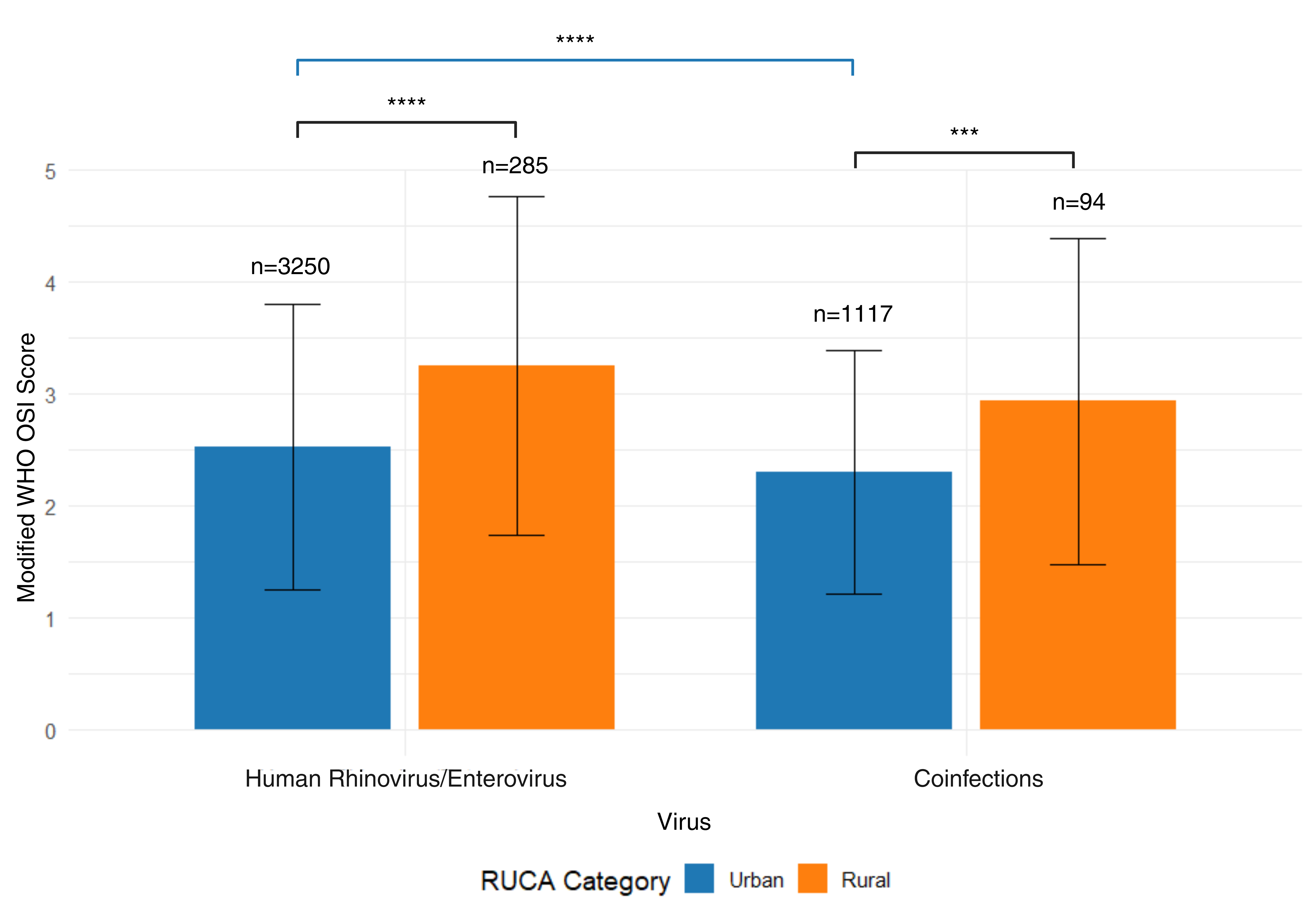


**Supplemental Figure 5.**


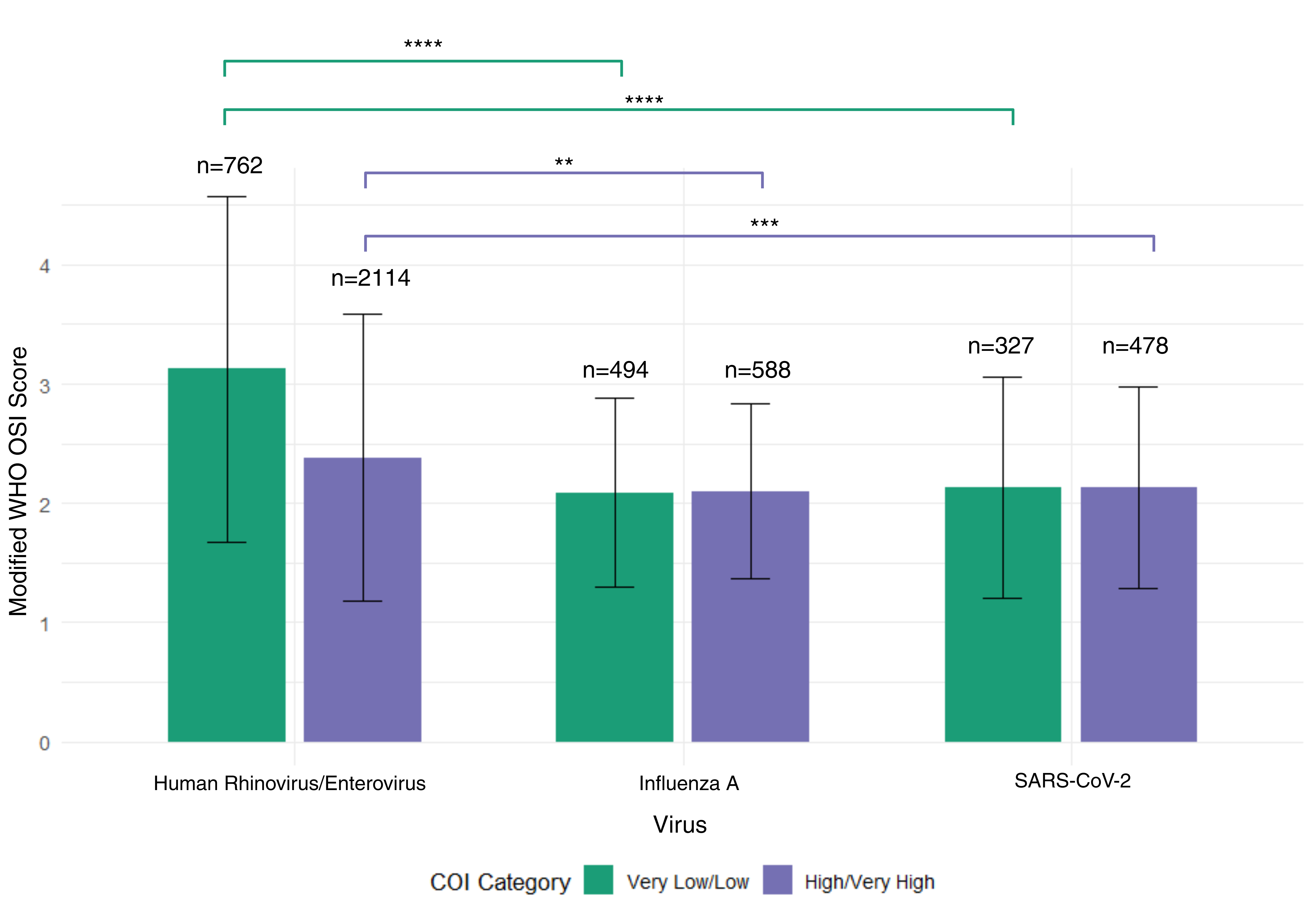

Supplement: Supplementary file 2 [file Supplementaryfile1.docx]
